# Genome-Wide Association Study Identifies Single Nucleotide Polymorphism in *DYRK1A* Associated with Replication of HIV-1 in Monocyte-Derived Macrophages

**DOI:** 10.1371/journal.pone.0017190

**Published:** 2011-02-25

**Authors:** Sebastiaan M. Bol, Perry D. Moerland, Sophie Limou, Yvonne van Remmerden, Cédric Coulonges, Daniëlle van Manen, Joshua T. Herbeck, Jacques Fellay, Margit Sieberer, Jantine G. Sietzema, Ruben van 't Slot, Jeremy Martinson, Jean-François Zagury, Hanneke Schuitemaker, Angélique B. van 't Wout

**Affiliations:** 1 Landsteiner Laboratory, Sanquin Research, Department of Experimental Immunology, and Center for Infection and Immunity Amsterdam (CINIMA) at the Academic Medical Center of the University of Amsterdam, Amsterdam, The Netherlands; 2 Bioinformatics Laboratory, Department of Clinical Epidemiology, Biostatistics and Bioinformatics, Academic Medical Center of the University of Amsterdam, The Netherlands; 3 Netherlands Bioinformatics Center (NBIC), Nijmegen, The Netherlands; 4 Chaire de Bioinformatique, Conservatoire National des Arts et Métiers, Paris, France; 5 Université Paris 12, INSERM U955, Paris, France; 6 Department of Microbiology, University of Washington School of Medicine, Seattle, Washington, United States of America; 7 Center for Human Genome Variation, Duke University, Durham, North Carolina, United States of America; 8 Complex Genetics Section, Department of Biomedical Genetics, University Medical Center Utrecht, Utrecht, The Netherlands; 9 Department of Human Genetics, University of Pittsburgh, Pittsburgh, Pennsylvania, United States of America; University of California, San Francisco, United States of America

## Abstract

**Background:**

HIV-1 infected macrophages play an important role in rendering resting T cells permissive for infection, in spreading HIV-1 to T cells, and in the pathogenesis of AIDS dementia. During highly active anti-retroviral treatment (HAART), macrophages keep producing virus because tissue penetration of antiretrovirals is suboptimal and the efficacy of some is reduced. Thus, to cure HIV-1 infection with antiretrovirals we will also need to efficiently inhibit viral replication in macrophages. The majority of the current drugs block the action of viral enzymes, whereas there is an abundance of yet unidentified host factors that could be targeted. We here present results from a genome-wide association study identifying novel genetic polymorphisms that affect *in vitro* HIV-1 replication in macrophages.

**Methodology/Principal Findings:**

Monocyte-derived macrophages from 393 blood donors were infected with HIV-1 and viral replication was determined using Gag p24 antigen levels. Genomic DNA from individuals with macrophages that had relatively low (n = 96) or high (n = 96) p24 production was used for SNP genotyping with the Illumina 610 Quad beadchip. A total of 494,656 SNPs that passed quality control were tested for association with HIV-1 replication in macrophages, using linear regression. We found a strong association between *in vitro* HIV-1 replication in monocyte-derived macrophages and SNP rs12483205 in *DYRK1A* (*p* = 2.16×10^−5^). While the association was not genome-wide significant (*p*<1×10^−7^), we could replicate this association using monocyte-derived macrophages from an independent group of 31 individuals (*p* = 0.0034). Combined analysis of the initial and replication cohort increased the strength of the association (*p* = 4.84×10^−6^). In addition, we found this SNP to be associated with HIV-1 disease progression *in vivo* in two independent cohort studies (*p* = 0.035 and *p* = 0.0048).

**Conclusions/Significance:**

These findings suggest that the kinase DYRK1A is involved in the replication of HIV-1, *in vitro* in macrophages as well as *in vivo*.

## Introduction

The development of highly active antiretroviral therapy (HAART) has been the biggest achievement in HIV/AIDS medicine. The current drug regimens can suppress plasma viral load to (near) undetectable levels. However, often residual levels of ongoing viremia can be detected [1], [2], even after treatment intensification [3], and complete eradication of HIV-1 from an infected patient has not been achieved with HAART [4]. HAART does not affect cells that are latently infected because in these cells the virus is not actively replicating. Activation of this viral reservoir of latently infected resting CD4^+^ T cells [5] and residual replication in monocytes/macrophages (reviewed in [6]) is believed to be responsible for the increase in plasma viral RNA levels that is observed after discontinuing HAART. Furthermore, cells residing in the gut associated lymphoid tissue (GALT) [7], [8], the testis [9], [10] and the central nervous system (CNS) [11], [12] may keep producing virus despite the use of antiretrovirals, because drug penetration of these tissues is suboptimal. As a result of this viral rebound and the existence of these sanctuary sites, HIV-1 infected individuals need to be on anti-HIV-1 medication for life. The emerging drug resistance [13], [14] and the severe side effects (reviewed by [15]), high costs and suboptimal tissue penetration of HAART necessitate transition from treatment to cure as soon as possible.

The viral reservoir is established early after primary HIV-1 infection [16], [17]. Although the precise mechanism is unknown, it has been shown that infected macrophages play a critical role in rendering resting CD4^+^ (but CCR5^−^) T cells permissive for HIV-1 infection [18]. Targeting these HIV-1 infected macrophages will not only help to eradicate the viral reservoir, but will also limit the spread of HIV-1 to CD4^+^ T cells [19], [20], prevent recruitment and activation of these cells [21], and reduce macrophage mediated apoptosis of T cells [22]–[25]. In addition, inhibiting or reducing HIV-1 replication in macrophages/microglia will be of importance in preventing the onset of AIDS dementia and other neurocognitive disorders associated with HIV-1 infection in the brain, because macrophages/microglia play a crucial role in the pathogenesis of these [26]–[28].

In the battle to cure HIV-1 infected individuals, identification of novel targets for the development of drugs that can be used to inhibit HIV-1 replication in macrophages will be required, especially since macrophages are relatively resistant to the cytopathic effect of HIV-1 replication [29]. Furthermore, tissue macrophages residing in the sanctuary sites described above are exposed to suboptimal levels of antiretrovirals, and the efficacy of protease inhibitors (PIs) and other late stage inhibitors in macrophages is substantially reduced [30], [31]. This lower efficacy of PIs in macrophages forms an obstacle in ending the ongoing residual replication, since only intervention of the virus' replication cycle at stages post reverse transcription and integration will prevent the production of new virions. Once the provirus is integrated in the host genome, integrase and reverse transcriptase inhibitors will no longer be effective in the cell.

Since the HIV-1 genome encodes only 15 proteins [32], it is dependent on numerous host proteins for its replication [33]. Intervention of the interaction between HIV-1 and these HIV-1 dependency factors (HDFs) can potentially be used to inhibit replication of the virus. Currently, the majority of drugs target viral enzymes: HIV-1 protease, reverse transcriptase and integrase (reviewed in [34]). The abundance of the HDFs offers great promise for finding drugs that may be less prone to emerging drug resistance of the virus, may better penetrate tissues and reach sanctuary sites, and may effectively prevent the formation of new virions in both monocytes/macrophages and CD4^+^ T cells. However, it will be crucial to find a balance between limiting the availability of HDFs and inhibiting viral replication, since most host proteins essential for HIV-1 replication may also play critical roles in the host cell.

Recently, a large number of host proteins that might be good targets for novel drugs to inhibit HIV-1 replication was identified [35]–[38]. These candidate proteins were found by measuring a significant change in HIV-1 replication after a genome-scale knock-down of host proteins, in different cell-lines. Since macrophages are important as reservoir and in sanctuary sites, it is also important to identify HDFs or host antiviral factors in these cells. Because primary macrophages are notoriously difficult to transfect and since HIV-1 replication in monocyte-derived macrophages (MDM) can vary enormously between donors [39]–[43] we decided to use a different approach and to actually exploit this genetic variation between individuals. When monocyte isolation, cell culture methods, medium and virus are all strictly controlled, remaining variation in HIV-1 replication observed in MDM from different donors can be assumed to be primarily of genetic origin. Finding an association between host genetic traits like a single nucleotide polymorphism (SNP) and HIV-1 replication in MDM would suggest that the locus tagged by this SNP is of importance for replication of HIV-1 in these cells. We therefore searched genome-wide for associations between SNPs and *in vitro* HIV-1 replication specifically in monocyte-derived macrophages.

## Results

### Association between single nucleotide polymorphism in the kinase *DYRK1A* and HIV-1 replication in monocyte-derived macrophages

**Figure 1:**
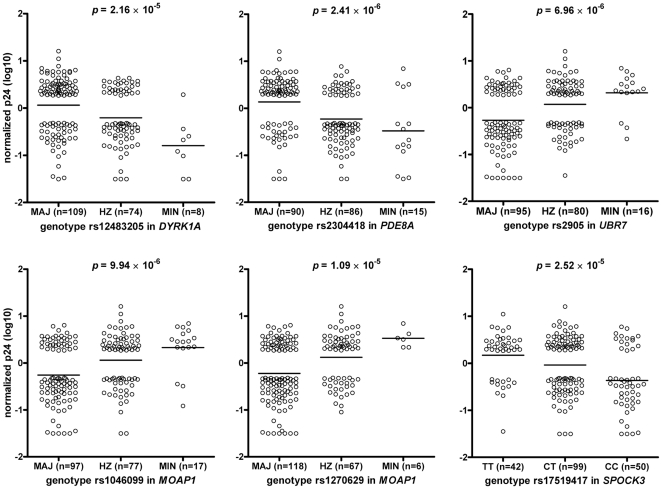
Association between HIV-1 replication in monocyte-derived macrophages (MDM) and the genotypes for the SNPs rs12483205 in *DYRK1A*, rs2304418 in *PDE8A*, rs2905 in *UBR7*, rs1046099 and rs1270629 in *MOAP1*, and rs17519417 in *SPOCK3*. Only donors with MDM with low (n = 95) or high (n = 96) HIV-1 replication *in vitro* were included in the genome-wide SNP analysis. This selection of donors with a more extreme phenotype explains the absence of circles in the middle section of the graphs. MAJ, homozygous for the major allele; HZ, heterozygote; MIN, homozygous for the minor allele.

A total of 494,656 SNPs passing quality control were tested for association with levels of HIV-1 replication in monocyte-derived macrophages (MDM) using linear regression. With data from 191 healthy blood donors whose MDM ranked in the bottom quartile with lowest (n = 95 donors) or top quartile with highest (n = 96 donors) Gag p24 production 14 days post infection with HIV-1 [39] (**Table 1**), we found strongest associations for SNPs in the genes *PDE8A* (rs2304418, *p* = 2.4×10^−6^ and rs12909130, *p* = 8.3×10^−6^), *UBR7* (rs2905, *p* = 7.0×10^−6^), *MOAP1* (rs1046099, *p* = 9.9×10^−6^ and rs1270629, *p* = 1.09×10^−5^), *DYRK1A* (rs12483205, *p* = 2.2×10^−5^) and *SPOCK3* (rs17519417, *p* = 2.5×10^−5^) (**Figure 1** **, Table S1**). The two SNPs in *PDE8A* were found to be in high linkage disequilibrium (LD; r^2^ = 0.97), whereas only a moderate degree of LD was found between rs1046099 and rs1270629 in *MOAP1* (r^2^ = 0.54) (**Table 2**). **Table 2** shows all other SNPs associated with HIV-1 replication in MDM (cutoff *p* value = 5×10^−5^; n = 16), and includes information about LD, location, number of donors homozygous for the minor allele (MIN) and empirical *p* values after permutation testing. Genotyping results for none of these 16 SNPs violated Hardy-Weinberg equilibrium.

**Table 1:** Characteristics of healthy donors whose macrophages showed either low, intermediate, or high HIV-1 replication *in vitro*.

	*In vitro* HIV-1 replication in monocyte-derived macrophages			
	Low	Intermediate	High	*p* value^a^
Donors (number)	95	202	96	n/a
Gender (males:females)	52∶43	unknown^b^	53∶43	0.948^c^
Age (years, mean ± SD)	46±11	49±11	47±12	0.371^d^
European ancestry (number, %)	95^e^, 100%	202^f^	96^e^, 100%	n/a
Normalized p24, log10 (mean ± SD)	−0.65±0.34	−0.01±0.16	0.49±0.18	<0.0001^d^

n/a, not applicable.

SD, standard deviation.

a *p* values only for comparison between donors with MDM that produced low levels of Gag p24 and donors with MDM that had high p24 production after infection with HIV-1.

b Information was not available.

c Pearson chi-square test.

d Two-sample t-test.

e Self-reported, Structure and Eigenstrat analysis (for details see Materials and Methods section).

f Self-reported only (for details see Materials and Methods section).

**Table 2:** SNPs (*p*<5×10^−5^) associated with *in vitro* HIV-1 replication in monocyte-derived macrophages.

	SNP	Closest gene	Location	Chr.	Position	*p* value	Empirical *p* *2	# MIN donors	Linkage disequilibrium (r^2^)*3			
1	rs2304418	*PDE8A*	Intronic	15	85640983	2.41×10^−6^	2.30×10^−6^	15	0.97▴			
2	rs2905	*UBR7*	3′ UTR	14	93693422	6.96×10^−6^	8.00×10^−6^	16		0.35▸	0.28▪	
3	rs12909130	*PDE8A*	Intronic	15	85590501	8.31×10^−6^	8.70×10^−6^	15	0.97▴			
4	rs1046099	*MOAP1*	3′ UTR	14	93649501	9.94×10^−6^	1.12×10^−5^	17			0.28▪	0.54•
5	rs1270629	*MOAP1*	Flanking 3′ UTR	14	93646409	1.09×10^−5^	1.15×10^−5^	6		0.35▸		0.54•
6	rs2828074		Intergenic	21	24710202	1.41×10^−5^	1.45×10^−5^	- *4				
7	rs12483205*1	*DYRK1A*	Intronic	21	38740824	2.16×10^−5^	2.26×10^−5^	8				
8	rs17519417	*SPOCK3*	Intronic	4	167659185	2.52×10^−5^	2.41×10^−5^	- *4				
9	rs16884060		Intergenic	5	10060297	3.20×10^−5^	3.12×10^−5^	4				
10	rs7856177	*SPTLC1*	Intronic	9	94839342	3.42×10^−5^	3.64×10^−5^	1		0.26▾		
11	rs8070997	*ACCN1*	Intronic	17	31428130	3.66×10^−5^	3.38×10^−5^	2				
12	rs1792745	*LOC642484*	Intronic	18	53804993	3.86×10^−5^	4.45×10^−5^	8				
13	rs7042102	*SPTLC1*	Flanking 3′ UTR	9	94763790	4.01×10^−5^	4.25×10^−5^	28	1.00◂	0.26▾		
14	rs10739923	*SPTLC1*	Flanking 3′ UTR	9	94746291	4.01×10^−5^	4.23×10^−5^	28	1.00◂			
15	rs12361072		Intergenic	11	23086211	4.93×10^−5^	5.46×10^−5^	34				1.00⧫
16	rs2468574		Intergenic	11	23096397	4.93×10^−5^	5.46×10^−5^	34				1.00⧫

Chr., chromosome.

*1 The effect of the SNP in *DYRK1A* was replicated with monocyte-derived macrophages from an independent group of 31 donors.

*2 Empirical *p* value calculated for linear regression using 10^7^ permutations of the genotypes.

*3 Measure for the magnitude of linkage disequilibrium (r^2^) between SNPs with identical symbols (▴, ◂, ▸, ▾, ▪, • or ⧫).

*4 Major genotype is the heterozygous genotype (∼50%); both classes of homozygous genotypes equally present (∼25% each).

**Figure 2:**
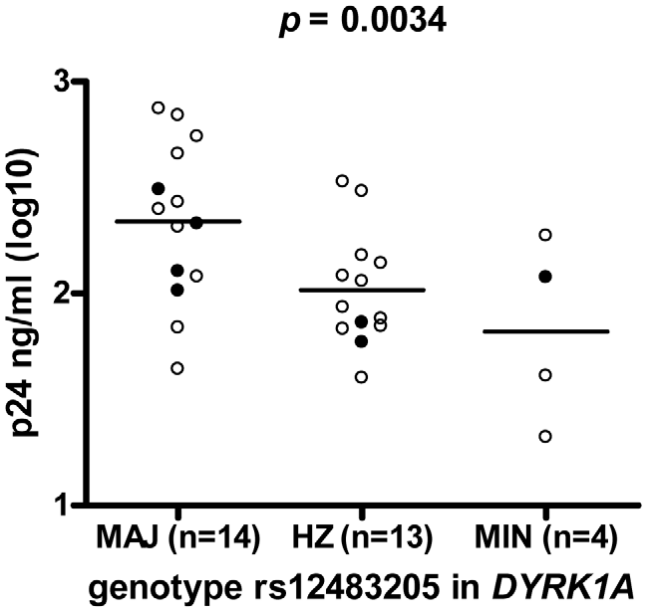
Significant association between rs12483205 and *in vitro* replication of HIV-1 in macrophages derived from an independent group of 31 healthy blood donors. The negative association between the rs12483205 minor allele and Gag p24 levels in MDM culture supernatant 14 days after inoculation with HIV-1, was found to match with the results from the genome-wide association study. Open circles represent results from donors with the *CCR5* Δ32 wild-type genotype, filled circles from donors with the *CCR5 wt/*Δ32 heterozygous genotype. MAJ, homozygous for the major allele; HZ, heterozygote; MIN, homozygous for the minor allele.

The empirical *p* values for linear regression using 10^7^ permutations of the genotype for each SNP in **Table 2** were in close agreement with the asymptotic *p* values (**Table 2**). However, none of these SNPs remained statistically significant after a conservative correction for multiple testing (Bonferroni threshold of *p*<1×10^−7^; **Figure S1**). We next investigated the association between the SNPs in the five most promising genes (rs2304418 in *PDE8A*, rs2905 in *UBR7*, rs1046099 and rs1270629 in *MOAP1*, rs12483205 in *DYRK1A* and rs17519417 in *SPOCK3*) in a second group of blood donors (replication cohort). MDM from an independent group of 32 healthy blood donors were infected with HIV-1 and Gag p24 levels were measured 14 days after infection. One donor was found to be homozygous for the 32 base pair deletion in *CCR5*, rendering the cells completely resistant to infection with CCR5-using HIV-1, and results obtained with macrophages from this donor were excluded from further analysis. The associations for the SNPs in *PDE8A*, *UBR7*, *MOAP1* and *SPOCK3* with Gag p24 production by MDM could not be replicated in this small group of 31 donors (data not shown). However, in this replication cohort we again found a strong association between SNP rs12483205 in *DYRK1A* and *in vitro* HIV-1 replication in macrophages (*p* = 0.0034) (**Figure 2** **, Table S2**), also after correction for cell number and normalization (*p* = 0.0081; n = 28, information on cell number was missing for 3 donors) (data not shown, **Table S2**). This association remained significant after correction for multiple testing (Bonferroni corrected *p* = 0.020 (0.0034×6 SNPs for which we tried to replicate the association)) and when calculating the empirical *p* value for linear regression using 10^5^ permutations of the genotypes (*p* = 0.004) (**Table S1**).

The *DYRK1A* SNP rs12483205 genotypes were then also determined for the 202 donors with MDM that had intermediate Gag p24 levels in the supernatant [39]. Adding results from this group of donors did not change the strength of the association between the rs12483205 genotype and HIV-1 replication in MDM (*p* = 2.09×10^−5^; n = 393) (**Figure S2A**, **Table S3**). As expected, combined analysis of both the initial group of donors (n = 393) and the replication cohort (n = 28) revealed an increase in the strength of the association for rs12483205 in *DYRK1A* (*p* = 4.84×10^−6^) (**Figure S2B**, **Table S4**).

### Effect of rs12483205 in *DYRK1A* is independent of the *CCR5* Δ32 genotype

The 32 base pair deletion in *CCR5* strongly affects replication of HIV-1, also in macrophages *in vitro* [39]. MDM from donors that were heterozygous for this 32 base pair deletion (n = 33) had significantly lower HIV-1 replication than donors without the deletion (n = 158) (*p* = 0.0036, two-sample t-test) (**Table S5**). There were significantly more *CCR5 wt/*Δ32 heterozygous donors present in the rs12483205 heterozygous (HZ) and rs12483205 MIN groups (*p* = 0.02, Fisher Exact test). After adjusting for the *CCR5 wt/*Δ32 heterozygous genotype by using it as a covariate in multivariate analysis, the strength of the association between the *DYRK1A* rs12483205 genotype and HIV-1 replication in MDM decreased (from *p* = 2.2×10^−5^ to *p* = 1.2×10^−4^), but persisted (**Table S5**). Moreover, the association of rs12483205 in *DYRK1A* with HIV-1 replication in MDM in the replication cohort of 31 donors, was independent of the *CCR5* Δ32 genotype (*p* = 0.0022, and *p* = 0.007 when corrected for cell number and normalized for date of isolation; **Table S2**), suggesting only a spurious relationship between the *CCR5* locus on chromosome 3 and SNP rs12483205 in *DYRK1A* located on chromosome 21. Indeed, this was supported by results from the multivariate analysis on the total group of 393 donors (*p* = 3.8×10^−5^, **Table S3**) and on the combined group of 421 (393+28 donors from the replication cohort) individuals (*p* = 9.7×10^−6^, **Table S4**).

### Minor variant of SNP rs12483205 in *DYRK1A* is associated with slower disease progression

Next, we investigated the association between HIV-1 load or disease course *in vivo* and the six most promising SNPs identified in our GWAS (rs12483205 in *DYRK1A*, rs2304418 in *PDE8A*, rs2905 in *UBR7*, rs1046099 and rs1270629 in *MOAP1* and rs17519417 in *SPOCK3*). Results from the Amsterdam Cohort Study (ACS) GWAS on HIV/AIDS disease progression (Van Manen *et al*., manuscript in preparation) and three previously published HIV-1 cohort studies with GWAS data were used: the CHAVI cohort [44], the GRIV [45], [46] and the MACS [47]. To avoid possible overlap between the replication cohorts, MACS samples were removed from the CHAVI cohort and the analysis was performed only using data from the European subset (EURO-CHAVI).

Illumina Human Hap BeadChips were used for genotyping DNA samples in the EURO-CHAVI cohort (550 K/1 M), the GRIV (300 K) and the ACS (300 K), whereas in the MACS they used Affymetrix Human 500 K GeneChips for the first stage discovery analysis [47]. Using different genotyping platforms results in genotype data for a different set of SNPs, which occasionally makes it necessary to estimate the unobserved genotypes, a process called SNP imputation [48]. When the imputation score (a measure for the reliability of the imputation) was below 0.8, imputation was considered unreliable and data was therefore not used. Results for SNPs rs2905 (*UBR7)*, rs1046099 (*MOAP1*), rs1270629 (*MOAP1*) and rs12483205 (*DYRK1A*) in the MACS, and rs1046099 (*MOAP1*) and rs17519417 (*SPOCK3*) in both the GRIV and the ACS, were based on SNP imputation. The imputation score for rs12483205 in *DYRK1A* was 0.94, and <0.8 for rs2905, rs1046099 and rs1270629. For rs1046099 and rs17519417 the imputation scores were 0.88 and 1 respectively, in the GRIV and in the ACS.

**Table 3** shows the results for association testing between disease progression or viral load and the identified SNPs in *DYRK1*, *PDE8A*, *UBR7*, *MOAP1* and *SPOCK3*. No significant associations or trends were found for the SNPs in *PDE8A*, *UBR7* or *MOAP1*. However, SNPs rs12483205 in *DYRK1A* and rs17519417 in SPOCK3 were significantly associated with *in vivo* endpoints in at least one of the four independent cohorts analyzed. While no association was observed in the GRIV and the ACS, we found an association between rs12483205 in *DYRK1A* and disease progression in the MACS (*p* = 0.0048, **Table 3**) and in the EURO-CHAVI cohort (*p* = 0.035, **Table 3**). In both cohort studies the minor allele of rs12483205 was associated with slower disease progression, which was consistent with the decreased *in vitro* replication of HIV-1 in MDM for the minor allele of rs12483205.

**Table 3:** Results (*p* values) for association testing using the additive analysis model, between disease progression or viral load and the SNPs in *DYRK1A*, *PDE8A*, *UBR7*, *MOAP1* and *SPOCK3*.

	EURO-CHAVI		GRIV		MACS		ACS	
SNP (gene)	viral load at set point	progression(CD4 T cells)	non-progressors - controls	rapid progressors - controls	rapid – moderate - non-progressors	AIDS(def. '87)	AIDS (def. '93)	AIDS related death
rs12483205 (*DYRK1A*)	>0.1	**0.035** *	>0.1	>0.1	**0.0048** * #	0.059* #	0.059	>0.1
rs2304418 (*PDE8A*)	>0.1	>0.1	>0.1	>0.1	>0.1	>0.1	>0.1	>0.1
rs2905 (*UBR7*)	>0.1	>0.1	>0.1	>0.1	N.A.	N.A.	>0.1	>0.1
rs1046099 (*MOAP1*)	>0.1	>0.1	>0.1	>0.1	N.A.	N.A.	0.075#	>0.1
rs1270629 (*MOAP1*)	>0.1	>0.1	>0.1	>0.1	N.A.	N.A.	>0.1	>0.1
rs17519417 (*SPOCK3*)	>0.1	>0.1	>0.1	>0.1	**0.012** * #	0.086* #	>0.1	>0.1

All *p* values <0.1 are shown.

*Effect of the minor allele consistent with our findings.

# Based on SNP imputation; imputation score >0.8.

N.A., not available (imputation score <0.8); EURO-CHAVI, European subset of the CHAVI cohort (n = 1,280 for viral load and n = 634 for disease progression) [44]; GRIV, Genomics of Resistance to Immunodeficiency Virus cohort (275 non-progressors vs. 1,352 controls [45], and 85 rapid progressors vs. 1,352 controls [46]); MACS, MultiCenter AIDS Cohort Study cohort (n = 156) [47]; ACS, Amsterdam Cohort Study (n = 404; Van Manen *et al*., manuscript in preparation).

Furthermore, there was an association between SNP genotype and progression to AIDS for rs17519417 in *SPOCK3* in the MACS [47] (*p* = 0.012, **Table 3**), with the C allele associated with slower progression. This was also in agreement with the association with lower *in vitro* HIV-1 replication in MDM in our study.

### SNP rs12483205 is localized near the 5′ untranslated region of *DYRK1A* transcript variant 3

**Figure 3:**
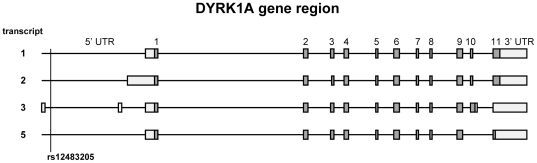
Schematic representation of the DYRK1A gene region, depicting all four transcript variants (1, 2, 3 and 5) and the localization of SNP rs12483205. Untranslated regions (UTR) are shown as open blocks, whereas exons are shown as filled blocks. SNP rs12483205 lies in close proximity to a part of the 5′ UTR unique for splice variant 3.

**Figure 4:**
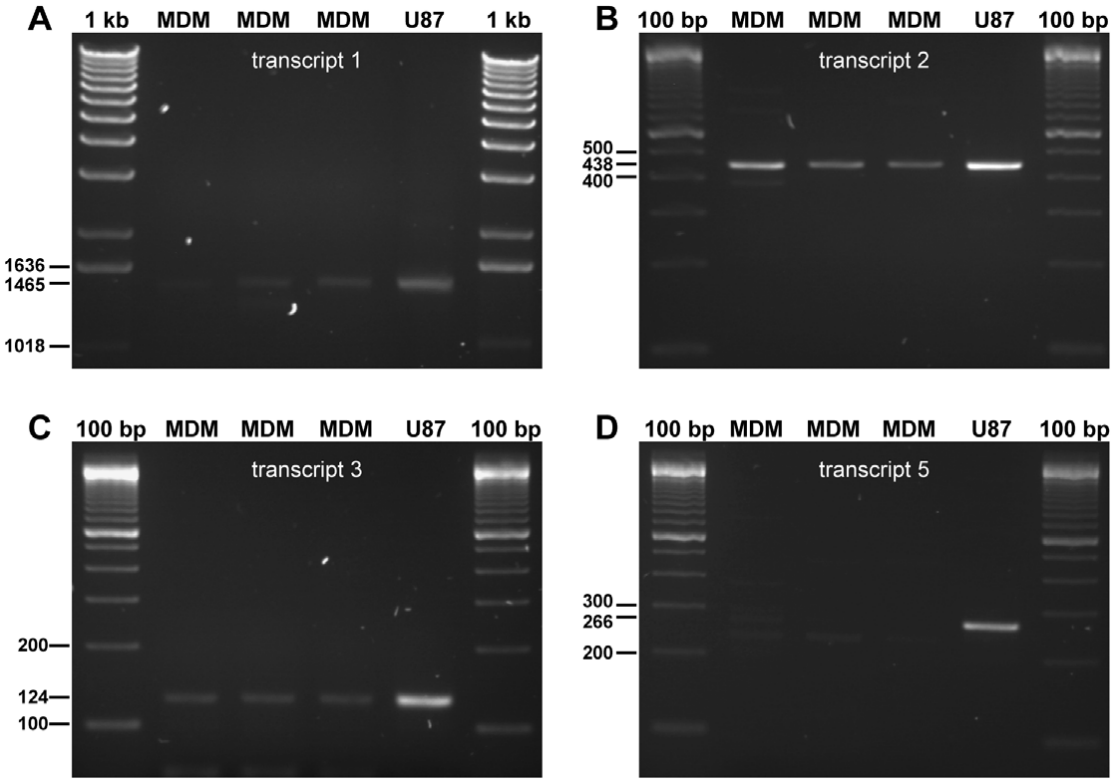
Detection of different *DYRK1A* transcript variants in macrophages. Amplicons were generated by PCR on cDNA from U87 cells, used as a positive control for the PCR, and from monocyte-derived macrophages (MDM) obtained from three individuals, using *DYRK1A* transcript variant specific primers. *DYRK1A* transcript variants 1, 2 and 3 were detected in MDM and U87 cells (panel A, B and C respectively). Transcript variant 5, however, was only convincingly detected in U87 cells, and not in MDM. Numbers on the left side of each picture indicate the size (in base pairs, bp) for the corresponding DNA fragment of the DNA ladder or PCR amplicons. 1 kb, 1 kb DNA ladder; 100 bp, 100 bp DNA ladder.

The *DYRK1A* mRNA has multiple splice forms. According to the NCBI Entrez Gene database [49] the alternative splicing can yield four different *DYRK1A* transcript variants, referred to as transcripts 1, 2, 3 and 5 (**Figure 3**). In addition to differences in the number or size of exons, there is also variation in the length of both the 5′ and 3′ untranslated region (UTR). Transcript variant 1 encodes the longest isoform (763 amino acids), which is a fraction larger than isoform 2 (754 amino acids). Isoforms 3 and 5 lack a C-terminal part of the full length protein (179 and 234 amino acids respectively), and consequently miss among others a poly-His domain. SNP rs12483205 in *DYRK1A* lies ∼900 base pairs downstream of the most distant 5′ UTR fragment of transcript variant 3 (**Figure 3**). PCR analysis with primers able to discriminate between all four known transcript variants (**Figure S3**) confirmed the presence of transcript variants 1, 2 and 3 in MDM, and in the glioma cell line U87 that was used as a positive control for the PCR (**Figure 4**). Transcript variant 5 was present in U87 cells, but was not detected in MDM (**Figure 4**). The DYRK1A gene region is devoid of known SNPs that are in high LD with rs12483205; currently available data on LD in this region does not reveal any SNP with an r^2^>0.5 in the Caucasian population [50]–[52].

## Discussion

Genome-wide studies provide a hypothesis free and unbiased approach to identify novel associations between genetic factors and the studied phenotype. Selecting donors with a more extreme *in vitro* phenotype allowed us to identify a number of promising SNPs in genes not previously linked to HIV-1, that are associated with replication levels of HIV-1 in monocyte-derived macrophages (MDM). Importantly, the association between rs12483205 in *DYRK1A* and *in vitro* HIV-1 replication could be replicated using MDM from an independent small group of donors, and was independent of the *CCR5* Δ32 genotype. Moreover, this SNP in *DYRK1A* was significantly associated with *in vivo* endpoints in two independent cohorts. SNPs in four other genes were not replicated in the small *in vitro* replication cohort, possibly reflecting insufficient power. However, the identified SNP in *SPOCK3* was replicated in one of the *in vivo* GWAS.

The use of, or even the dependence on, certain cellular factors by HIV-1 can differ between the various target cells such as T cells and macrophages. For example, the cellular factors GATA-3, ETS-1 and NF-ATc are T cell specific [53], [54], whereas C/EBPβ is required for HIV-1 replication in macrophages, but not in T cells [55], [56]. To test whether our top SNPs were macrophage-specific, we investigated if these SNPs were also associated with HIV-1 replication in CD4^+^ T cells. Genotyping DNA from 128 blood donors for whom the level of HIV-1 replication in PHA-stimulated CD4^+^ T cells was previously determined [57], revealed no association between the level of HIV-1 replication in T cells and the genotype of the five SNPs tested. This finding could indeed be an indication that the effects of some of the SNPs that we have identified are macrophage-specific, but also could reflect lack of power (n = 128, and not only extreme phenotypes). The finding that the SNP rs12483205 in *DYRK1A* was associated with *in vivo* endpoints could indicate a role for HIV-1 infection of macrophages in disease progression.

DYRK1A is a kinase that phosphorylates serine and threonine residues. The gene encoding for this kinase is located on the Down syndrome critical region on chromosome 21 which is thought to be responsible for the features of Down syndrome. For this reason, the emphasis of DYRK1A research has been on its role in trisomy 21 and neurological dysfunction [58]–[63]. Substrates for DYRK1A comprise among many others the transcription factors CREB [64], FKHR [65], GLI1 [66], NFAT [67], [68] and STAT3 [69]. This large number of protein interactions and substrates described for DYRK1A strongly suggests it plays an important role in the regulation of gene expression and cell function. While the molecular mechanisms underlying the effect of DYRK1A on HIV-1 replication in MDM remain to be unraveled, the critical role of kinases in general for the replication of HIV-1 in macrophages has already been established [70]–[75].

Both SNPs in *MOAP1* that we found to be associated with HIV-1 replication in MDM are in close proximity to the UBR7 gene region (44 and 47 kb for rs1046099 and rs1270629 respectively), yet the degree of LD is fairly low (r^2^ = 0.28 and 0.35 for rs1046099 and rs1270629 respectively) which could indicate that the observed associations for the SNPs in *UBR7* and *MOAP1* with HIV-1 replication are independent of each other. MOAP1 is a homodimeric protein that binds both proapoptotic (BAX) and prosurvival (BCL2) molecules [76]. MOAP1 has no known direct effect on HIV-1 replication. However, it is well recognized that HIV-1 infection affects the regulation of programmed cell death (reviewed in [77]) and that host factors induce apoptosis during the infection process to prevent viral dissemination [78], [79]. This host factor-induced apoptosis was found to be associated with reduced HIV-1 replication in MDM [78], [79]. Only very little is known about UBR7. However, studies on other members of the UBR family support the hypothesis that UBR7 is be associated with HIV-1 replication. Proteins of the UBR family are E3 ligases that recognize degradation signals at the N-terminus of proteins (N-degrons) and conjugate ubiquitin to the target protein [80]. The HIV-1 protein integrase contains an N-terminal phenylalanine that is recognized as a degradation signal by UBR1, UBR2 and UBR4 [80], [81] and indeed, these proteins control the level of HIV-1 integrase [80]. Knocking down UBR1, UBR2 and UBR4 resulted in higher levels of HIV-1 integrase [80], yet inhibition of the proteasome resulted in a further increase of HIV-1 integrase [80], suggesting the presence of other E3 ligases that target N-degrons of HIV-1 integrase. The results of our study could indicate that UBR7 affects HIV-1 replication in MDM through regulation of HIV-1 integrase.

In addition to the SNP in *DYRK1A* we also found associations for rs17519417 in *SPOCK3* with time to progression to AIDS. SPOCK3, also referred to as Testican 3, is a proteoglycan and one of the components of the extracellular matrix. It inhibits processing of metalloproteinase 2 (MMP-2) [82]. There are indications that MMP-2 might play a role in the pathogenesis of AIDS-related Kaposi's sarcoma [83], [84] or HIV-1 associated dementia [85]–[87].

While we could not replicate the GWAS result for the SNP in *PDE8A*, results from other *in vitro* studies strongly suggest that PDE8A plays an important role in the replication cycle of HIV-1. Phosphodiesterase 8A (PDE8A) specifically hydrolyses cAMP to AMP. Previous studies that showed strong inhibition of HIV-1 replication after efficient knock-down [38], interaction between HIV-1 Tat protein and PDE8A [88], [89] and the deleterious effect of high levels of cAMP on HIV-1 replication [90], all profoundly suggest that this type of phosphodiesterase is indeed important for *in vitro* HIV-1 replication.

To further validate our findings it will be important to also investigate the role of the identified SNPs and genes in HIV-1 related pathologies that are more specifically affected by macrophages, such as HIV-1 associated dementia and AIDS related lymphomas [91]. Here, the contribution of macrophages could be more eminent than in cohorts that study time to progression to AIDS, which might be more T cell dependent, especially since sometimes AIDS is defined by CD4^+^ T cell counts.

Follow-up experiments that will identify the causal SNP and study its effect on the primary protein structure, protein folding, alternative splicing or expression (level, localization and timing) will be essential to better understand the precise mechanism by which this SNP affects HIV-1 replication in MDM. This will be a valuable step that might help determine if, and to what extent the host protein can be efficiently targeted by novel antiretroviral drugs. Finding a SNP in a gene encoding the kinase DYRK1A that affects HIV-1 replication, holds great promise for finding molecules to be safely used as potentially novel anti-HIV-1 medication [92].

## Materials and Methods

### Ethics statement

This study has been conducted in accordance with the ethical principles set out in the declaration of Helsinki, written informed consent was obtained from all participants and was approved by the Medical Ethics Committee of the Academic Medical Center in Amsterdam and the Ethics Advisory Body of the Sanquin Blood Supply Foundation, The Netherlands.

### Study population

We previously determined the ability of HIV-1 to replicate in monocyte-derived macrophages (MDM) from 429 different healthy seronegative blood donors [39]. In brief, Gag p24 levels were measured in MDM culture supernatant 14 days post infection by an in-house enzyme-linked immunosorbent assay. To correct for differences in the number of viable MDM present at day 14 post infection, p24 levels were expressed per 10,000 cells. Since monocyte isolations were performed in four time frames and by two operators, p24 levels were normalized by dividing through the median per period and operator. These normalized p24 levels were subsequently used as a measure for *in vitro* HIV-1 replication in MDM.

After excluding donors that were homozygous for the 32 base pair deletion in *CCR5* (n = 5) or not from European decent (n = 30), we selected 192 individuals whose MDM gave the highest (n = 96) or the lowest (n = 96) p24 production *in vitro*, for SNP genotyping; thus representing two groups of donors with MDM that had a more extreme phenotype (**Table 1**). Inclusion of donors with extreme phenotypes is known to increase power in genetic association studies [93]. These two groups with a more extreme phenotype were separated by a group with intermediate HIV-1 replication in MDM which consisted of 202 donors. There was no difference in age or distribution of males and females between the group with MDM that had low HIV-1 replication and the group with MDM that had high Gag p24 production (*p* = 0.95, Pearson chi-square test and *p* = 0.37, two sample t-test, respectively; **Table 1**). One donor from the group with low *in vitro* HIV replication in MDM was excluded for further analysis because the corresponding DNA samples did not pass the quality control (SNP call rate <0.98; see paragraph “Quality control of SNP genotyping data” below).

Cells (monocytes and lymphocytes) used to replicate the genome-wide association study (GWAS) findings were derived from blood samples collected from an independent group of 32 healthy blood donors (replication cohort).

### Genotyping

Peripheral blood lymphocytes (PBL) from each donor were used for DNA isolation using the QIAamp “DNA blood mini kit” (Qiagen, Valencia, CA, USA). For the two groups of donors whose MDM had highest (n = 96 donors) or lowest (n = 96 donors) *in vitro* HIV Gag p24 production, we used the Illumina Infinium Human Hap 610-Quad BeadChip (Illumina, San Diego, CA, USA) [94] for SNP genotyping. ABI TaqMan® SNP genotyping assays were used to genotype DNA from the replication cohort for rs12483205 (C_31609775_10), rs12909130 (C_1342209_10) as a proxy for rs2304418, rs2905 (C_3236245_20), rs1046099 (C_7585751_10) and rs17519417 (C_33238869_10) (Applied Biosystems, Carlsbad, CA, USA). All TaqMan® assays were run on a LightCycler® 480 system (Roche, Basel, Switzerland) using the following amplification cycles: 10 min 95°C; 50 cycles of 15 sec 95°C, 1 min 60°C.

### Quality control of SNP genotyping data

One donor DNA sample did not pass the minimal genotyping call rate threshold of 98% and this sample was excluded for further SNP association analysis. The Illumina 610Q SNP bead chip contained 620,901 markers to detect common genetic variation, including 21,890 markers for copy number variation (CNV). After excluding a total of 126,245 SNPs (minor allele frequency <0.05, or SNP call frequency <0.98, or SNPs for which there were no donors homozygous for the minor allele) and copy number variation markers, 494,656 SNPs were left to be tested for association between genotype and *in vitro* HIV-1 replication MDM. Violation of Hardy-Weinberg equilibrium was assessed post-analysis for all SNPs with *p*<5×10^−5^. Gender calls in BeadStudio (version 3.1.3.0) were compared with self-reported gender and were found to match for all donors (n = 191).

### Identification of population stratification

Donors who reported that either one or more parents or grand parents were born outside of Europe had been excluded from further analysis (as described in the paragraph “Study population” above). The genetic homogeneity of the genotyped donors whose MDM gave lowest (n = 96) or highest (n = 96) Gag p24 production *in vitro* was confirmed by both Structure [95] and Eigenstrat [96] analysis. As expected, the Q-Q plot displaying the normality of the *p* value distribution did not show deviations from what is expected under the null hypothesis (λ = 1.0024; λ = 1.0 is expected with a null hypothesis; **Figure S4**), indicating little effect of population stratification.

### Statistical analysis

We set a lower detection limit (background signal) for the normalized p24 values based on the highest results that we found for a donor that was homozygous for the 32 base pair deletion in *CCR5*. Normalized p24 values were log10 transformed to allow for parametric testing.

Association was tested assuming an additive relationship between the genetic variants (wild-type, heterozygous and homozygous for the minor allele) and phenotype, using linear regression. Permutation testing was done to empirically determine the corresponding *p* value based on the observed linear regression F-statistic relative to the distribution of the F-statistic estimated from 10^7^ permutations of the genotypes. All R code and data used for the calculations can be found as supplementary online material (**Tables S1, S2, S3, S4 and S5** and text file “**R Scripts S1**”).

Linkage disequilibrium (LD) between SNPs was calculated using data obtained from genotyping DNA from our studied population (n = 191) with the Illumina SNP chip, using Haploview software (version 4.2) [50]. SNP genotype imputations for our study population were performed using Impute software [48] and the Caucasian HapMap population (release 21) as the reference panel.

### *DYRK1A* transcript detection in monocyte-derived macrophages

Seven day old MDM and U87 cells were used for RNA isolation and subsequent cDNA synthesis using the Roche “high pure RNA isolation” and “transcriptor first strand cDNA synthesis” kit (Roche, Basel, Switzerland). To uniquely detect the presence of transcript variants 1, 2, 3 and 5, we used primer pairs 5′-TGATATTGTCATGTTACAGAGGCGG-3′ (forward) and 5′-CTGACGCACCTGGGGACTG-3′ (reverse), 5′-TGTCTCTGAGGTTCTTTTCCAGTG-3′ (forward) and 5′-AGCACCCTCTCAATTCCCAATGCC-3′ (reverse), 5′-GCTCGCACGTGGTTCATTTGCT-3′ (forward) and 5′-TCCTTAGACAGGAACGTCATGAACCT-3′ (reverse), and 5′-CAGGAGGACCTGGTGGGCGA-3′ (forward) and 5′-TGCTGACGCACCTGAGCTTG-3′ (reverse) respectively (**Figure S3**). For the detection of transcript variants 1, 2, 3 and 5 the following amplification cycles were used: 2 min 95°C; 35 cycles of 30 sec 95°C, 30 sec 62°C (64°C for transcript 1), 30 sec 72°C (90 sec for transcript 1); 10 min 72°C.

## Supporting Information

Figure S1 Manhattan plot displaying the -log *p* value of the association for 494,656 SNPs tested with *in vitro* replication of HIV-1 in monocyte-derived macrophages. Signals are seen for SNPs in chromosome 14 (SNPs in *UBR7* or *MOAP1*), 15 (SNPs in *PDE8A*) and 21 (SNP rs12483205 in *DYRK1A*, and intergenic SNP rs2828074 >14 Mb upstream of rs12483205). The threshold for genome-wide significance is -log *p*>7 (dashed line). The plot was created using the WGA viewer software version 1.26G [97].
(TIF)

Figure S2 Association between HIV-1 replication in monocyte-derived macrophages (MDM) and the SNP rs12483205 genotype in the gene *DYRK1A*, for the total group of 393 blood donors (A), and this group of donors joined with donors from the replication cohort for which we had normalized data (n = 28; total n = 421) (B). DNA from donors with MDM that had low (n = 95) or high (n = 96) HIV-1 replication *in vitro* was used for the genome-wide association screen. Inclusion of genotype and normalized p24 data from donors with MDM that had intermediate Gag p24 production did not change the strength of the association (*p* = 2.09×10^−5^), whereas combining the initial group of donors (n = 393) and the replication cohort (n = 28) increased the strength of the association (*p* = 4.84×10^−6^). MAJ, homozygous for the major allele; HZ, heterozygote; MIN, homozygous for the minor allele.
(TIF)

Figure S3 Schematic representation of the alignment of all four known *DYRK1A* mRNAs. Primers are depicted as arrows and were used to amplify a unique region for each of the transcripts. The start and stop codons are shown as green and red vertical lines respectively.
(TIF)

Figure S4 Q-Q plot showing the expected and observed distribution of the *p* values. The line and the corresponding Lambda (λ) suggest there are no systematic differences in allele frequencies between subpopulations in our study population due to differences in genetic background of donors. The plot was created using the WGA viewer software version 1.26G [97].
(TIF)

Table S1 (TXT)

Table S2 (TXT)

Table S3 (TXT)

Table S4 (TXT)

Table S5 (TXT)

R Scripts S1 (TXT)
