# Supplementary figures and images for: Genome-Wide Association Study Identifies Single Nucleotide Polymorphism in DYRK1A Associated with Replication of HIV-1 in Monocyte-Derived Macrophages
Source: PLoS One. 2011 Feb 25;6(2):e17190. doi: 10.1371/journal.pone.0017190 (PMC3045405; doi:10.1371/journal.pone.0017190)

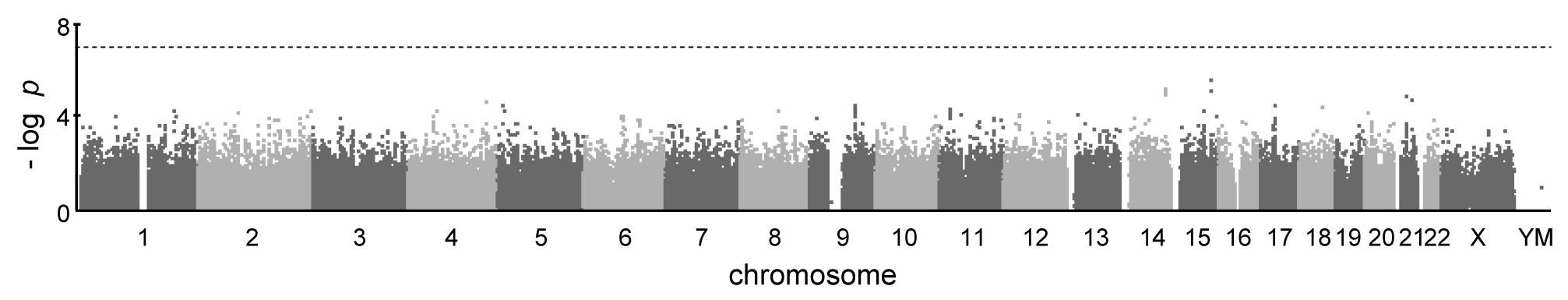

Supplement: Figure S1 — Manhattan plot displaying the -log p value of the association for 494,656 SNPs tested with in vitro replication of HIV-1 in monocyte-derived macrophages. Signals are seen for SNPs in chromosome 14 (SNPs in UBR7 or MOAP1), 15 (SNPs in PDE8A) and 21 (SNP rs12483205 in DYRK1A, and intergenic SNP rs2828074 >14 Mb upstream of rs12483205). The threshold for genome-wide significance is -log p>7 (dashed line). The plot was created using the WGA viewer software version 1.26G [97]. (TIF) [file pone.0017190.s001.tif]

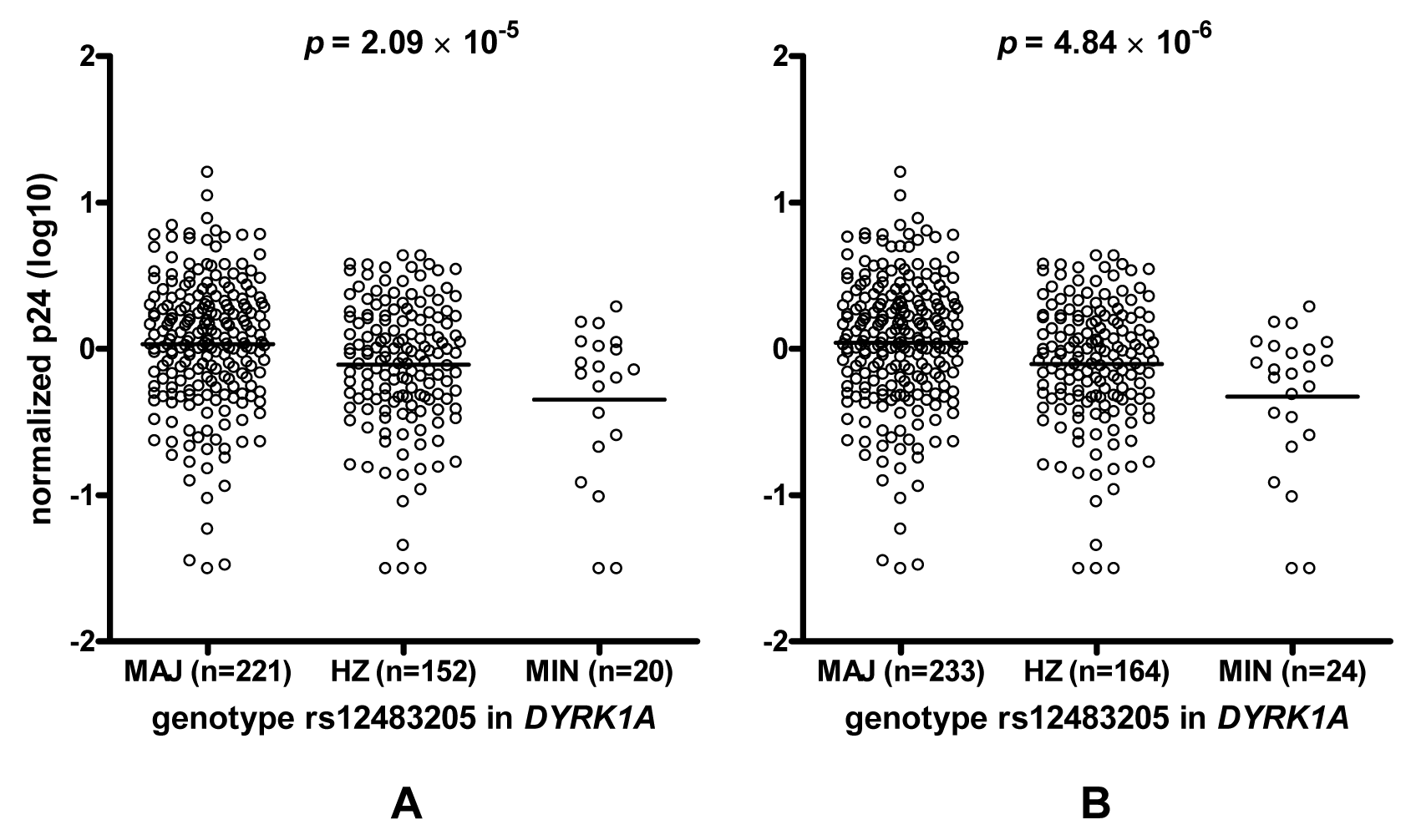

Supplement: Figure S2 — Association between HIV-1 replication in monocyte-derived macrophages (MDM) and the SNP rs12483205 genotype in the gene DYRK1A, for the total group of 393 blood donors (A), and this group of donors joined with donors from the replication cohort for which we had normalized data (n = 28; total n = 421) (B). DNA from donors with MDM that had low (n = 95) or high (n = 96) HIV-1 replication in vitro was used for the genome-wide association screen. Inclusion of genotype and normalized p24 data from donors with MDM that had intermediate Gag p24 production did not change the strength of the association (p = 2.09×10^−5^), whereas combining the initial group of donors (n = 393) and the replication cohort (n = 28) increased the strength of the association (p = 4.84×10^−6^). MAJ, homozygous for the major allele; HZ, heterozygote; MIN, homozygous for the minor allele. (TIF) [file pone.0017190.s002.tif]

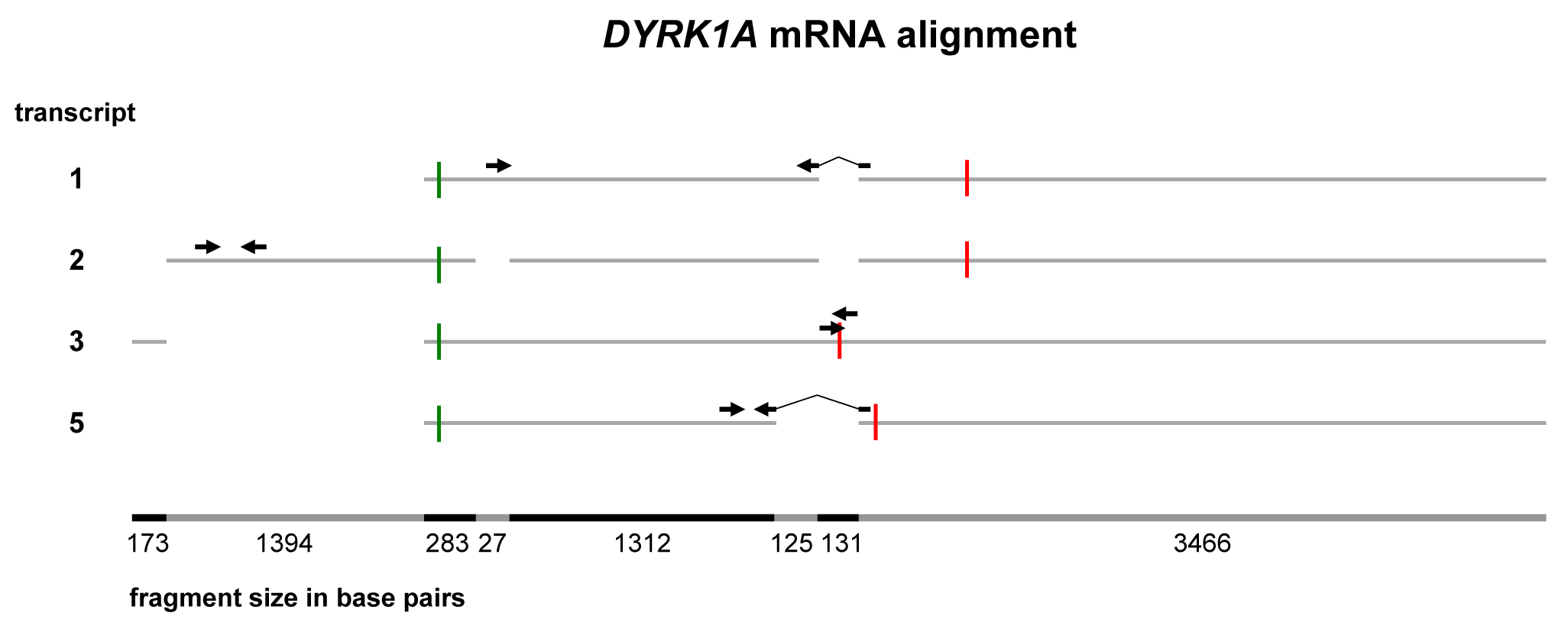

Supplement: Figure S3 — Schematic representation of the alignment of all four known DYRK1A mRNAs. Primers are depicted as arrows and were used to amplify a unique region for each of the transcripts. The start and stop codons are shown as green and red vertical lines respectively. (TIF) [file pone.0017190.s003.tif]

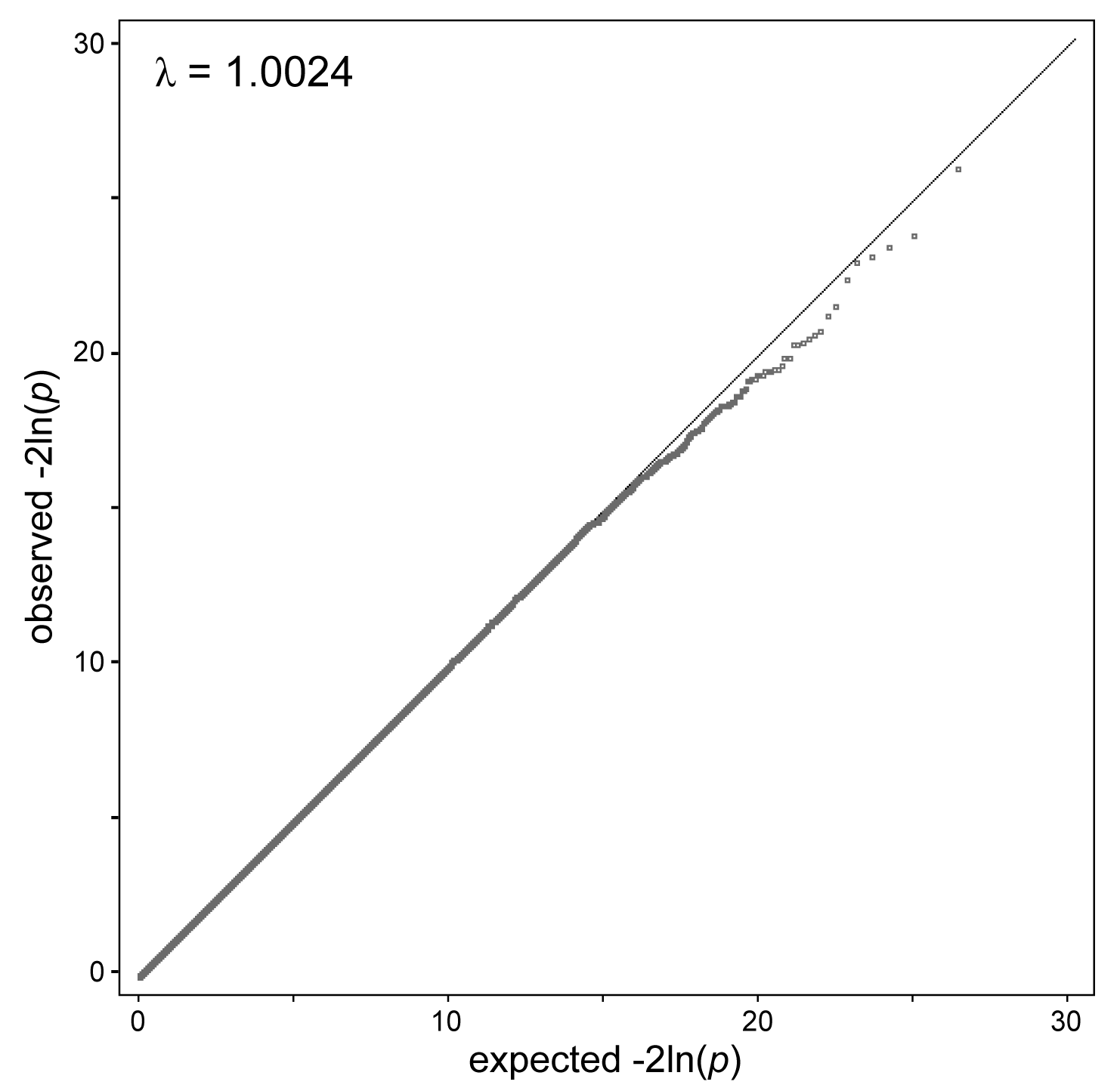

Supplement: Figure S4 — Q-Q plot showing the expected and observed distribution of the p values. The line and the corresponding Lambda (λ) suggest there are no systematic differences in allele frequencies between subpopulations in our study population due to differences in genetic background of donors. The plot was created using the WGA viewer software version 1.26G [97]. (TIF) [file pone.0017190.s004.tif]
